# The Etiology of Anemia Among Pregnant Women in the Hill State of Himachal Pradesh in North India: A Cross-Sectional Study

**DOI:** 10.7759/cureus.21444

**Published:** 2022-01-20

**Authors:** Sushruti Kaushal, Tanu Priya, Sunil Thakur, Poojan Marwaha, Harpreet Kaur

**Affiliations:** 1 Obstetrics and Gynaecology, All India Institute of Medical Sciences, Bilaspur, IND; 2 Obstetrics and Gynaecology, Dr. Rajendra Prasad Government Medical College, Kangra, IND; 3 Anesthesiology, Dr. Radhakrishnan Government Medical College, Hamirpur, IND; 4 Obstetrics and Gynaecology, Shri Lal Bahadur Shastri Government Medical College, Mandi, IND

**Keywords:** hemoglobinopathies, folate, ferritin, pregnant, anemia

## Abstract

Background and objective

Anemia during pregnancy is a major cause of maternal and fetal complications including mortality. A study of the etiology of anemia is required to formulate guidelines for the prevention and treatment of the condition. To this end, we conducted a study among anemic women in northern India.

Materials and methods

A cross-sectional study was conducted among anemic antenatal women attending the outpatient department at a tertiary care hospital in Himachal Pradesh, India, involving 172 participants. Complete blood count, serum ferritin level, serum B12, serum folate levels, high-performance liquid chromatography (HPLC), liver function tests, and renal function tests were performed.

Results

The mean hemoglobin level among the subjects was 8.87 g/dl with a standard deviation of 0.79; 50% of women had serum ferritin levels of less than 15 ng/ml, 48.8% had serum B12 levels of less than 150 pg/ml. and 33.72% of women had serum folate levels of less than 3 ng/ml. Of note, 13.37% of women had either low or deficient levels for all three parameters; 14 women had abnormal results on HPLC. All nutrient deficiencies (ferritin, folate, and vitamin B12) were found in all morphological types of anemia. Significantly, 73.26% of iron-deficient anemic women had additional folate or vitamin B12 deficiencies, suggesting that additional methods would be required to decrease the prevalence of anemia. Two-thirds of the women in our study were vegetarians, a contributing factor towards a high percentage of vitamin B12 deficiency among women. ß-thalassemia trait was the most common abnormality found, consistent with the high prevalence of ß-thalassemia in north India.

Conclusion

Multiple deficiencies should be treated simultaneously in anemic women. Vitamin B12 deficiency is an important contributor to anemia, in addition to iron and folate deficiency.

## Introduction

Anemia among pregnant women is a public health problem of global concern. Anemia during pregnancy is a major cause of maternal and fetal complications including mortality [1]. It decreases women’s ability to withstand bleeding before and after delivery and makes them more prone to infections. The World Health Organization (WHO) estimates that 1,15,000 maternal deaths globally are attributable to iron-deficiency anemia annually [2]. Fetal complications include prematurity, low birth weight, intrauterine growth restriction, and child mortality [1].

In India, anemia during pregnancy is a significant public health problem, with 45.7% of pregnant women in urban areas and 52.1% in rural areas having hemoglobin levels <11 g/dl [3]. Anemia is the underlying cause or contributing factor for 20-40% of maternal deaths in India, which account for 80% of maternal deaths attributable to anemia in South Asia [2].

Iron deficiency is believed to be the most important cause of anemia. Approximately 50% of anemia in pregnant women is believed to be due to iron deficiency [4], but the proportion probably varies among population groups and in different areas, according to local conditions [4]. Other important causes include micronutrient deficiencies (e.g., vitamin B12 and folate) and inherited disorders that affect hemoglobin synthesis and red blood cell survival, e.g., hemoglobinopathies.

Studies on the etiology of anemia have been scarce, probably due to its complex etiology and a lack of diagnostic facilities. Global estimates of the prevalence of iron deficiency are based on trials studying the effects of treatment [4]. Direct investigation into the etiology of anemia is completely lacking in India and in our state of Himachal Pradesh. Any strategy implemented to prevent and treat anemia should be tailored to local conditions, taking into account the specific etiology and prevalence of anemia in a given setting and population group. Therefore, it is essential to study the etiology of anemia during pregnancy to formulate realistic guidelines for the treatment and prevention of the condition during pregnancy.

## Materials and methods

This cross-sectional observational study was conducted among anemic antenatal women attending the outpatient department at a tertiary care hospital in the state of Himachal Pradesh in India over a period of one year. Anemia was defined as a hemoglobin level of less than 11 g/dL, as per the existing WHO definition of anemia [5]. The sample size was calculated based on the WHO estimates of 50% of anemia being attributable to iron deficiency [4] with an 80% level of confidence and 5% margin of error. The calculated sample size was 164. We planned to recruit 180 participants, after adjusting for loss to follow-up. We eventually conducted the study among 172 participants.

All pregnant women with anemia were considered for recruitment irrespective of the gestational age. Women with underlying chronic diseases like chronic kidney disease and those with a history of blood transfusion in the previous year were excluded from the study. The women were recruited after obtaining written informed consent in the patients’ native language. Demographic data, like age, educational qualifications, and socioeconomic status, were obtained and recorded on a pre-designed and pre-tested case record form. Women were interviewed for their obstetric history and examined for general and obstetric parameters.

The investigations performed on these women were as follows: complete blood count with peripheral smear, serum ferritin level as the marker of iron status, serum B12, and serum folate levels. Serum ferritin concentrations were determined using an immunoenzymometric assay. Serum ferritin levels less than 30 ng/mL were considered low and those less than 15 ng/mL were considered as the cutoff for iron deficiency [6]. Vitamin B12 deficiency was diagnosed if serum B12 levels were less than 150 ng/L while levels between 150-200 ng/L were considered as borderline deficiency. Folate deficiency was defined as serum folate levels of less than 3 ng/mL while values between 3-4.5 ng/mL were considered low levels but indeterminate to diagnose folate deficiency [7]. High-performance liquid chromatography (HPLC) was done as a diagnostic test for hemoglobinopathies. Additional investigations like renal and hepatic function tests, card tests for viral markers, and urine microscopy were done to rule out underlying chronic diseases. None of the participants had any signs/symptoms or investigative reports suggestive of HIV or any other chronic illness. As the study area is located at a high altitude with rarely reported cases of malaria, participants were not investigated for malaria as a cause of anemia [8].

Women were managed as per standard protocols and bone marrow aspiration/biopsy was decided to be performed among those who needed it as per standard guidelines. None of the women in our study required bone marrow aspiration/biopsy.

Statistical analysis

Percentages were calculated for the sociodemographic variables as well as for the distribution of various micronutrient deficiencies in the anemic women using Microsoft Excel version 16.51 (Microsoft Corporation, Redmond, WA). The median and range of variables that were not distributed normally were calculated using Graphpad Prism 9 software (GraphPad Software Inc., San Diego, CA). The study was approved by the Institutional Ethics Committee of the SLBS Government Medical College, Mandi at Nerchowk.

## Results

Clinical and investigative data were collected for 172 anemic pregnant women. Of these, 5.8% were in their first trimester, 20.93% were in the second trimester, and 73.25% were in the third trimester of pregnancy. The distribution of various sociodemographic factors among the participants is presented in Table 1.

**Table 1:** Sociodemographic characteristics of the participants *Modified Kuppuswamy Scale

Sociodemographic variables	Values
Age in years, mean (±SD)	26.20 (4.61)
BMI, kg/m^2^, mean (±SD)	25.6 (4.4)
Underweight, BMI <18.5, n (%)	4 (2.32)
Normal weight, BMI 18.5-24.9, n (%)	76 (44.18)
Overweight, BMI 25-29.9, n (%)	70 (40.69)
Obese, BMI ≥30, n (%)	22 (12.79)
Residence, n (%)	
Rural	130 (75.58)
Urban	42 (24.41)
Profession, n (%)	
Homemaker	162 (94.18)
Other	10 (5.81)
Dietary habits, n (%)	
Vegetarian	115 (66.86)
Nonvegetarian	57 (33.13)
Socioeconomic status (SES)*, n (%)	
Low SES	97 (56.39)
Middle SES	73 (42.44)
Upper-middle SES	2 (1.16)
Parity, n (%)	
0	61 (35.40)
1	76 (44.18)
2	33 (19.18)
3	2 (1.16)
Abortions, n (%)	
0	152 (88.37)
1	12 (6.97)
2	6 (3.48)
3	0 (0)
4	2 (1.16)
Duration of pregnancy, n (%)	
First trimester	10 (5.8)
Second trimester	36 (20.93)
Third trimester	126 (73.25)
Adequate antenatal visits (as per gestation), n (%)	
Yes	166 (96.51)
No	6 (3.48)
Deworming done in pregnancy (in women >16 weeks), n=156, n (%)	
Yes	38 (24.35)
No	118 (75.64)
Pre-conceptional folic acid intake, n (%)	
Yes	12 (6.97)
No	160 (93.02)
First-trimester folic acid intake, n (%)	
Yes	136 (79.06)
No	36 (20.93)

The mean hemoglobin level among women in our study was 8.87 g/dL with a standard deviation of 0.79. Out of 172 participants, most of the women had mild (50%) or moderate (48.8%) anemia while only two (1.16%) had severe anemia. The results of the hematological and biochemical analyses of the participants are presented in Table 2.

**Table 2:** Hematologic and biochemical analyses of the participants TIBC: total iron-binding capacity

Variables	Median	Minimum	Maximum	N
Hemoglobin (g/dL)	8.95	5.4	10.0	172
Total leucocyte count (thou/µl)	8750	4200	1780	172
Platelet count (x 10^3^/microL)	232	98	463	172
Ferritin (ng/mL)	14.7	4	412	172
Serum iron (µg/dL)	48	19	426	172
TIBC (µg/dL)	590.5	341	853	172
Transferrin saturation (%)	8.65	3.58	100.6	172
Serum transferrin (mg/dL)	495.5	252	831	172
Serum B12 (ng/L)	151.5	67	478	172
Serum folate (ng/mL)	4.2	1.00	50.00	172

Details

Iron Deficiency

Out of 172 anemic women studied, 86 (50%) had serum ferritin levels of less than 15 ng/mL. Another 54 (31.40%) women had serum ferritin levels between 15-30 ng/mL, indicating an iron-deficient state.

Vitamin B12 Deficiency

Among the participants, 84 (48.8%) women had serum B12 levels of less than 150 ng/L and 32 (18.60%) women had B12 values between 150-200 ng/L. Overall, 116 (67.44%) women had either serum B12 deficiency or had borderline low levels.

Folate Deficiency

In our cohort, 58 (33.72%) women had serum folate levels of less than 3 ng/mL while 34 (19.77%) women had folate levels between 3-4.5 ng/mL. All pregnant women in India are prescribed folic acid supplementation during pregnancy as per a national program. These low folate levels indicate non-compliance with the taking of nutritional supplements in addition to poor dietary intake.

Concomitant Deficiencies

Among the participants, three (1.74%) women had deficient levels for serum ferritin, serum B12, and serum folate while 23 (13.37%) women had either low or deficient levels of all three. Concomitant deficiencies are presented in Table 3.

**Table 3:** Distribution of micronutrient deficiencies by iron status *Iron-deficiency: S. ferritin <15 ng/mL. **Folate deficiency: S. folate <3.0 µg/L. ***Vitamin B12 deficiency: S. vitamin B12 <150 ng/L

Other deficiency	Iron-deficient*, n (%)	Not iron-deficient, n (%)
None	28 (31.8)	22 (26.19)
Folate**	24 (27.27)	14 (15.90)
Vitamin B12***	30 (34.09)	34 (38.64)
Folate + vitamin B12	6 (6.81)	14 (15.90)
Total	88	84

Hemoglobinopathies

In our cohort, 14 women had abnormal results on analysis for hemoglobinopathies; 10 women had ß-thalassemia trait, one woman had homozygous HbE disease, two had HbJ variant trait, and one had HbE variant trait.

Morphological classification of anemia and its relation with nutrient deficiencies

Anemia was classified as normocytic if the mean corpuscular volume (MCV) was 79-93.3 fl. If MCV was <79 fl, it was classified as microcytic anemia, and MCV >93.3 fl was classified as macrocytic anemia [9]. Classification of anemia and various nutrient deficiencies found in women with microcytic, normocytic, and macrocytic anemia are presented in Table 4.

**Table 4:** Distribution of micronutrient deficiencies with the morphological classification of anemia

Type of anemia	S. ferritin <30 ng/mL, n (%)	S. ferritin <15 ng/mL, n (%)	S. vitamin B12 <200 ng/L, n (%)	S. vitamin B12 <150 ng/L, n (%)	S. folate <4.5 µg/L, n (%)	S. folate <3.0 µg/L, n (%)
Microcytic anemia (n=86)	76 (88.37%)	58 (67.44%)	56 (65.11%)	36 (41.86%)	42 (48.84%)	22 (25.58%)
Normocytic anemia (n=58)	30 (51.72%)	20 (34.48%)	38 (67.86%)	28 (50%)	28 (50%)	8 (14.29%)
Macrocytic anemia (n=28)	16 (57.14%)	8 (28.57%)	12 (42.86%)	10 (35.71%)	14 (50%)	8 (28.57%)

## Discussion

Anemia during pregnancy is a major public health problem with 40.1% of pregnant women worldwide suffering from the condition [1]. This prevalence is even higher among pregnant women in India, with 50% of pregnant women suffering from anemia [1]. Nutritional disorders and chronic infections are the leading causes of anemia during pregnancy. Among the nutritional deficiencies, iron deficiency is estimated to be the most common nutrient deficiency worldwide. Folate deficiency, vitamin B12 deficiency, helminthic infections, and inherited disorders are the other important contributors to anemia in pregnancy. About two-thirds of the women in our study were vegetarians, which constitutes a risk group for vitamin B12 deficiency, especially during pregnancy [7].

Helminthic infections

The WHO recommends a single dose of albendazole to be given to pregnant women after the first trimester of pregnancy, in areas where anemia among pregnant women is more than 40% [10]. National guidelines in India also recommend deworming in the second trimester of pregnancy. Despite this, only 24.35% of the eligible women in our study had received albendazole during pregnancy. This suggests that better coverage of pregnant women in this deworming program could contribute towards the reduction of anemia in pregnant women in our population.

Iron-deficiency anemia

In our study, we found that 81.3% of women were iron-deficient with serum ferritin levels of less than 30 ng/mL while 50% had serum ferritin levels of less than 15 ng/mL. This is comparable to worldwide estimates of 50% anemia attributable to iron deficiency [11]. We could not find any studies that reported the prevalence of iron deficiency among anemic pregnant women. A study from another state in northern India found iron deficiency (serum ferritin <15 ng/mL) in 67.7% of pregnant women [12]. Another hospital-based study in north India reported low serum ferritin levels in 56.6% of pregnant women [13]. Contributing factors towards the high prevalence of iron deficiency may include poor compliance with oral iron therapy, poor absorption of oral iron, and untreated helminthic infections. We recommend the wider use of injectable iron preparations at a lower threshold, which would circumvent the problems of poor compliance and poor absorption.

We also found that 73.26% of iron-deficient anemic women had additional nutrient deficiencies in the form of folate deficiency, vitamin B12 deficiency, or both, indicating that additional methods (besides iron supplementation) would be required to decrease the prevalence of anemia.

Folate deficiency

Diagnosis of folate deficiency in pregnancy is complicated by multiple factors. Cutoffs for diagnosis outside of pregnancy are <3 µg/L for deficiency and 3-4.5 µg/L is considered to be indeterminate zone [7]. Hemodilution during pregnancy, especially in later months, may lead to falsely low levels. Requirement of folate supplements during pregnancy and preferential delivery of folate to the fetus may precipitate severe maternal deficiency. This could be further aggravated in the presence of pregnancy-related complications like multiple pregnancies and hyperemesis. Although routine supplementation of folic acid is recommended in the pre-conceptional period and during pregnancy, 93.02% of women had not taken folic acid in the pre-conceptional period while 20.93% had not taken folic acid in the first trimester of pregnancy.

Since cutoff levels to diagnose deficiency have not been established separately for pregnant women, we decided to err on the side of more and to use the levels similar to those for the non-pregnant population. Of note, 48.83% of women in our study had serum folate levels in the indeterminate zone while 22.09% had folate deficiency. Folate deficiency in our study was slightly lower than that reported by Pathak et al. in 2007 (26.3%) [12]. Earlier studies in India had shown folate deficiency in the range of 21-30% [14-16].

Vitamin B12 deficiency

In our study, 61.62% of the anemic pregnant women had serum vitamin B12 levels of less than 200 ng/L. Two-thirds of the women in our study were vegetarians, which could be a contributing factor towards vitamin B12 deficiency in such a high percentage of women. A study done in another state in the region found vitamin B12 deficiency in 74.1% of pregnant women.

Concomitant deficiencies

In our cohort, 60.46% of women had more than one nutritional deficiency while 26.7% had low levels or deficiency of all three parameters. We are not aware of any studies in the literature that have analyzed multiple deficiencies in pregnant women and hence cannot compare our data with older studies.

Hemoglobinopathies

Thalassemia is the most prevalent hemoglobinopathy in India, with the average frequency of carriers being 3-4% [17]. In our study, we found that 8.1% of women had abnormal results on testing for hemoglobinopathy. ß-thalassemia trait was the most common abnormality found. This is consistent with the fact that parts of north India have a high prevalence of ß-thalassemia [17].

Morphological classification of anemia

As shown in Table 4, we found that all nutrient deficiencies (ferritin, folate, and vitamin B12) were present in all morphological types of anemia, i.e., microcytic, normocytic, and macrocytic anemias. Traditionally, microcytic anemia is treated with iron supplementation, macrocytic anemia with folate and vitamin B12 supplementation, and dimorphic anemia with iron, folate, and vitamin B12. In this study, morphological classification of anemia correlated poorly with nutrient deficiencies. Although we had patients with deficiency of iron as well as folate/vitamin B12, dimorphic anemia was not reported in a single case. Treating patients solely on the basis of morphological classification could lead to inadequate or partial treatment.

Our study was conducted in the setting of a tertiary care hospital in the outpatient department. The findings of our study may not be representative of the general antenatal population, with a higher proportion of referred and complicated cases. Likewise, patients presenting with very severe anemia with complications usually present to the emergency and are likely to have a different etiological distribution.

## Conclusions

This study provides an insight into the various causes of anemia during pregnancy. The findings of our study can contribute to the formulation of strategies for the prevention and treatment of anemia during pregnancy. We found that a large proportion of women have multiple nutrient deficiencies, instead of just one. This could be attributed to poor dietary intake of multiple nutrients. We would like to emphasize the importance of treating these multiple deficiencies simultaneously in anemic women. Our findings also suggest that vitamin B12 deficiency is an important contributor towards anemia in our population, which is predominantly vegetarian, in addition to iron and folate deficiency.
